# Tetranectin as a Potential Biomarker Associated with Post-Operative Inotropic Support Following the Norwood Procedure in Neonates: A Pilot Study

**DOI:** 10.3390/jcdd13080373

**Published:** 2026-08-06

**Authors:** Ananya Manchikalapati, Namasivayam Ambalavanan, AKM F. Rahman, Inmaculada Aban, Brian A. Halloran, Kristal M. Hock, Michele Kong, Santiago Borasino, Ahmed Asfari

**Affiliations:** 1 Department of Pediatrics, Division of Critical Care, University of Texas Southwestern Medical Center, Dallas, TX 75390, USA; 2 Department of Pediatrics, Division of Neonatology, University of Alabama at Birmingham, Birmingham, AL 35294, USA; nambalav@uabmc.edu (N.A.); bahalloran@uabmc.edu (B.A.H.); 3 Department of Biostatistics, University of Alabama at Birmingham, Birmingham, AL 35294, USA; frahman@uab.edu (A.F.R.); caban@uab.edu (I.A.); 4 Department of Pediatrics, Division of Cardiology, Section of Cardiac Critical Care, University of Alabama at Birmingham, Birmingham, AL 35294, USA; kristalhock@uabmc.edu (K.M.H.); sborasino@uabmc.edu (S.B.); 5 Department of Pediatrics, Division of Critical Care, University of Alabama at Birmingham, Birmingham, AL 35294, USA; mkong@uabmc.edu; 6 Department of Pediatrics, Division of Cardiology, University of Texas Southwestern Medical Center, Dallas, TX 75390, USA; ahmed.asfari@utsouthwestern.edu

**Keywords:** low cardiac output state, biomarker, Norwood procedure, Tetranectin, cardiac surgery

## Abstract

Background: Tetranectin (TN) is a protein that plays a role in tissue remodeling. In adults with heart failure, TN has a better prognostic value compared to B-type natriuretic peptide (BNP). We aimed to examine changes in TN in neonates following the Norwood operation and explore its prognostic value. Methods: Retrospective observational pilot cohort study in a tertiary pediatric cardiac intensive care unit. Neonates who underwent the Norwood procedure and had stored plasma samples in our biorepository were screened for inclusion. TN and BNP levels were measured by ELISA at five different time points in relation to cardiopulmonary bypass (CPB) in 26 neonates who met the inclusion criteria. The primary outcomes were time to lactate clearance and duration of inotropic support. Results: Univariate analysis showed that higher TN levels at 0 and 4 h were associated with a longer duration of inotropic support. Logistic regression analyses comparing TN levels at different time points with duration of inotropic support showed that TN level at 4 h post-CPB was a significant predictor (*p* = 0.04) for a longer duration of inotropic support with an AUC of 0.876. Conclusions: In this pilot cohort, higher observed TN concentrations in the early post-operative period were associated with longer duration of inotropic support. TN was not found to be significantly associated with time to lactate clearance. These exploratory findings require validation in larger prospective studies before TN can be considered for clinical prognostication.

## 1. Introduction

Low cardiac output syndrome (LCOS) is frequently encountered after cardiac surgery utilizing cardiopulmonary bypass (CPB). Prior studies reported association of LCOS with increased morbidity, mortality, and healthcare resource utilization [1,2]. Although LCOS can be transient after cardiac surgery, it can result in reduced oxygen delivery (DO_2_) and subsequent tissue hypoxia and end organ injury. LCOS is most commonly defined as a decrease in cardiac index (CI) to <2.0L/min/m^2^ in conjunction with signs of tissue hypoperfusion [1,3]. LCOS after heart surgery may be induced by several pathologic processes triggered by CPB. CPB induces endothelial cell injury and activates an immune and inflammatory response [4,5]. For infants undergoing the Norwood procedure, there are added disadvantages due to the needed aortic cross clamp, cardiac circulatory arrest, and deep hypothermia [6,7]. Early identification of LCOS is crucial to enable goal-directed therapy and improve clinical outcomes.

Identification of specific serum biological molecules as diagnostic or prognostic biomarkers for LCOS could aid in earlier identification and therapeutic decision making. There have been various clinical and laboratory findings that have been used to identify LCOS following cardiac surgery in neonates. At the bedside, clinical metrics such as serum lactate, central venous oxygen saturation (S_c_VO_2_), regional near-infrared spectroscopy (NIRS), urine output, and inotropic support are often used as bedside surrogates of a patient’s cardiac output. These metrics can be influenced by multiple patient and management factors.

Various laboratory biomarkers such as B-type natriuretic peptide (BNP), N-terminal-pro-BNP (NT-proBNP) and Troponin I (cTn-I) have been studied as prognostic indicators of heart failure and LCOS [8,9,10,11,12]. BNP and NT-proBNP remain amongst the most extensively studied biomarkers in pediatric cardiac surgery and are frequently used as indicators of ventricular dysfunction and post-operative hemodynamic compromise. Previous studies have demonstrated associations between elevated BNP concentration and adverse outcomes following congenital heart surgery [13,14]. Furthermore, the combination of BNP with other biomarkers has been reported to improve risk stratification compared with BNP alone [15]. However, there is no validated stand-alone biomarker that is reflective of LCOS. Despite the low level of evidence and lack of validation, BNP and NT-proBNP are used in evaluating the stages of heart failure after Stage I palliation. The continued search for novel biomarkers therefore remains important, particularly in high-risk populations such as neonates undergoing the Norwood procedure.

Tetranectin (TN), a novel protein thought to have a role in tissue remodeling, has been studied in adults with heart failure and found to have a higher diagnostic specificity and sensitivity than BNP [16]. Other adult studies have investigated its profile following CPB [17] but there have been no studies looking at the characteristics of this protein in the neonatal and pediatric population. TN may play a role in cardiomyocyte protection after cardiac surgery utilizing CPB. Based on findings from our prior unbiased proteomic analysis for patients with cardiac surgery [18], we hypothesized that serum TN concentration and its trend can be used for early identification of LCOS in pediatric patients. To test this hypothesis, we evaluated TN serum concentrations in a relatively homogenous sample of neonates with hypoplastic left heart syndrome (HLHS) following Norwood operation. We measured serial TN concentrations and examined their correlation with lactate clearance and duration of post-operative inotropic support.

## 2. Materials and Methods

### 2.1. Patient Selection

This study was reviewed and approved by the University of Alabama at Birmingham’s Institutional Review Board (IRB) (approval number IRB-300007319, approved 21 September 2021). Informed consent was waived. All study procedures were followed in accordance with the ethical standards of the University of Alabama at Birmingham Institutional Review Board and with the Helsinki Declaration of 1975, as most recently amended. At Children’s of Alabama under a separate IRB approval, all patients < 7 yo who are scheduled for cardiac surgery are approached for participation in the congenital heart center biorepository. Subjects for our study were selected from this biorepository. Patients who underwent Norwood operation were screened for inclusion.

### 2.2. Study Population

In this pilot study, we measured TN in a cohort of patients with a homogenous pathophysiology and age but at high risk of developing LCOS with variable severity. We selected neonates (<29 days old) who had single ventricle physiology who underwent the Norwood procedure. These neonates are known to be at high risk of LCOS.

The inclusion criteria were (1) neonates who underwent the Norwood procedure and (2) neonates who had available serum samples at the five time points before and after surgery in the biorepository. We had a total of 71 neonates who underwent the Norwood procedure over a seven-year period (July 2015 to July 2021), of whom 29 had available samples at the five time points. Amongst those who met the predefined inclusion criteria (*n* = 29), we excluded (1) neonates who required extracorporeal membrane oxygenation (ECMO) intraoperatively or within 48 h post-operative (*n* = 2) as patient’s volume of distribution can be altered by ECMO support and (2) premature neonates with gestational age < 37 weeks (*n* = 1) to ensure homogenous patients’ sample. In the final patient cohort who underwent further analysis (*n* = 26), 23 had HLHS and three had HLHS variant.

### 2.3. Data Definition and Collection

Data for the patients included were collected from the electronic health record at the time of initial hospitalization. The primary outcomes were time to lactate clearance and duration of post-operative inotropic support. These two outcomes are used at bedside as markers for LCOS. Time to lactate clearance was defined as the time (in hours) to first lactate levels ≤ 2.0 mmol/L after returning from the operating room (OR) post-operatively. Post-operative vasoactive management followed our institutional Norwood pathway. Patients routinely return to the cardiac ICU on milrinone infusion at 0.5 mcg/kg/min and epinephrine at 0.02–0.04 mcg/kg/min, with stress-dose hydrocortisone per institutional protocol. Escalations of epinephrine and additional vasopressin or norepinephrine were individualized according to blood pressure, bedside hemodynamic assessment and clinical markers of systemic perfusion such as urine output, NIRS, and ScVO_2_ in addition to lactate trends. Duration of inotropic support was the time in hours that vasopressors were required post-operatively excluding milrinone and calcium drips as these are used routinely in the post-operative care for our patients with Norwood operation in our heart center.

Demographic data collected included sex, race (self-reported), age at surgery, weight, cardiac physiology, and type of surgery. Clinical data collected included duration of open sternum, need for re-intervention defined as either diagnostic or interventional catheterization study or surgical intervention, need for ECMO after 48 h post-operatively, hospital length of stay (HLOS), length of mechanical ventilation (MV) (time in hours until the first successful extubation of at least 48 h), duration of non-invasive ventilation, duration of CPB, aortic cross clamp time, number of CPB runs, duration of deep hypothermic circulatory arrest, vasoactive inotropic support (VIS) score [19] at all five time points, renal NIRS at all five time points, percent fluid overload and duration of renal replacement therapy. Additional biochemical data collected included S_c_VO_2_, lactate levels, arterial pH, partial pressure of carbon dioxide (pCO_2_), partial pressure of oxygen in arterial blood (PaCO_2_), blood urea nitrogen (BUN), and creatinine at all five time points of interest.

### 2.4. Plasma Samples

Blood samples before and after CPB were collected by bedside nurses in heparinized tubes. Subsequently, plasma (supernatant) was isolated by centrifugation and then stored in the heart center biorepository at −80 °C. Plasma samples were studied at five time points: pre-CPB, 0 h post-CPB, 4 h post-CPB, 24 h post-CPB, and 48 h post-CPB.

### 2.5. Analysis of Samples for BNP and TN

BNP in plasma samples was diluted by a factor of 15 and was quantified using a Human NPPB (BNP) ELISA kit (Invitrogen EHNPPB, Waltham, MA, USA) following the manufacturer’s instructions. Quantitation of TN in the serum samples required a 1000-fold dilution for accurate estimation using a Human TN ELISA kit (Abcam ab213832, Cambridge, UK).

### 2.6. Statistical Analysis

Descriptive analyses (medians, interquartile range (IQR) for non-normal continuous measure and frequency distributions (5) for categorical measures) were used to describe patient demographic and clinical characteristics outcomes as appropriate. Survival Weibull regression analyses were performed for the two primary outcomes, time to lactate clearance and duration of inotropic support, to identify the potential risk factors associated with them. In addition, we divided our cohort based on the duration of inotropic support into tertiles and compared the lower (*n* = 9) and upper tertile (*n* = 9) subjects for the outcome duration of inotropic support using logistic regression. To quantify the predictability of TN at different hours to the outcome, we produced the receiver operating characteristic (ROC). All hypothesis tests were two-tailed, using a *p* < 0.05 to indicate statistical significance. We performed analyses in SAS for Windows version 9.4 (SAS Institute, Cary, NC, USA).

## 3. Results

### 3.1. Primary Clinical Outcomes

Of the 26 neonates who underwent the Norwood procedure, the median CPB duration was 142 min (IQR 133, 164) with a median aortic cross clamp duration of 58.8 min (IQR 53.7, 71.0). The median duration of inotropic support was 91.6 h (IQR 71.9, 144.3) and the median time to lactate clearance was 25 h (IQR 16.8, 41.7). The survival to hospital discharge was 92%. Other clinical outcomes and variables are listed in Table 1.

### 3.2. Serial TN and BNP Levels

TN and BNP levels were measured at each time point (Table 2). The mean BNP levels changed from the pre-op value at 0 h post-CPB (6.27 ± 5.8 ng/mL) to peak at 4 h post-CPB (14.73 ± 6.9 ng/mL) with a subsequent downtrend. The mean TN levels demonstrated an increase from pre-op value until 4 h post-CPB (6.42 ± 2.4 mcg/mL) followed by a subsequent downtrend.

### 3.3. Secondary Clinical Variables Related to LCOS

We studied various clinical markers as indicators of LCOS (Table 3). Peak lactate levels were noted at 0 h post-CPB [median 7.8 (6.6, 9.8)] which coincided with the peak in the VIS scores [median 14.15 (9.0, 16.3)], with a downtrend over the remaining 48 h following CPB. NIRS and ScVO_2_ demonstrated a fairly stable trend throughout the period of observation. Our cohort had a positive fluid overload % (4.88 ± 6.7) in the first 24 h post-Norwood procedure which improved by a second post-op day with a negative fluid overload % by 48 h (−1.35 ± 3.6). Our standard post-op care for patients undergoing the Norwood procedure includes the intraoperative placement and utilization of peritoneal dialysis in the 48–72 h following surgery.

### 3.4. TN as a Predictor of Duration of Inotropic Support

We evaluated the association between TN levels, BNP levels and other clinical indices to our primary outcomes. The univariate analysis showed that higher TN levels at 0 and 4 h post-CPB were associated with a longer duration of inotropic support. After adjustment for BNP levels, higher TN levels at 0 h and 4 h post-CPB were independently associated with longer duration of inotropic support but not found to be significantly associated with time to lactate clearance (Table 4). In contrast, BNP levels post-CPB were not significantly associated with duration of inotropic support.

**Figure 1:**
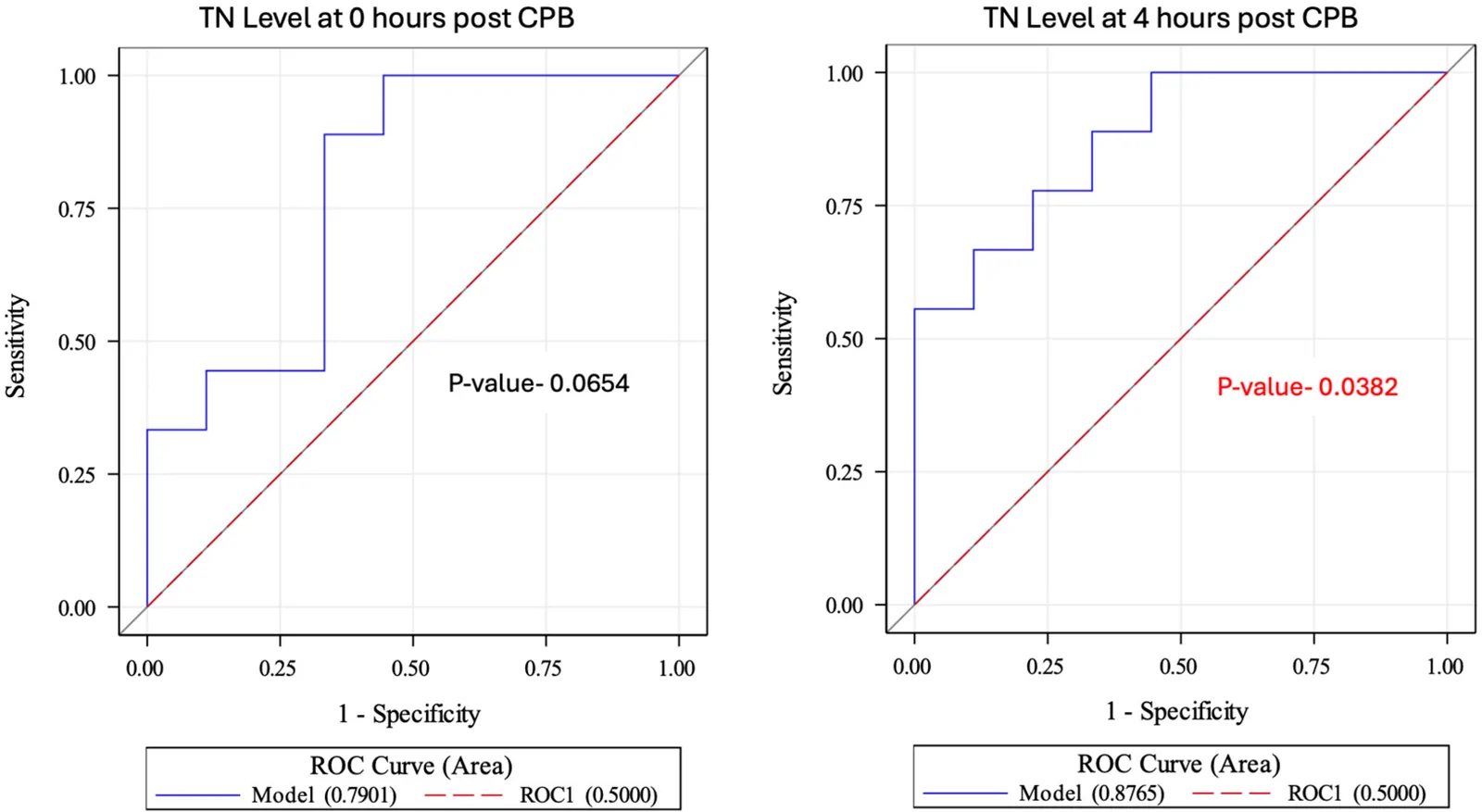
Exploratory ROC analysis of TN concentrations at 0 and 4 h post-CPB for distinguishing neonates in the upper vs. lower tertile of post-operative inotropic support duration.

Separate logistic regression analyses comparing TN levels at different time points with duration of inotropic support showed that TN levels at 4 h post-CPB was a significant predictor (*p* = 0.0382) for a longer duration of inotropic support with an AUC of 0.876 (Figure 1).

**Figure 2:**
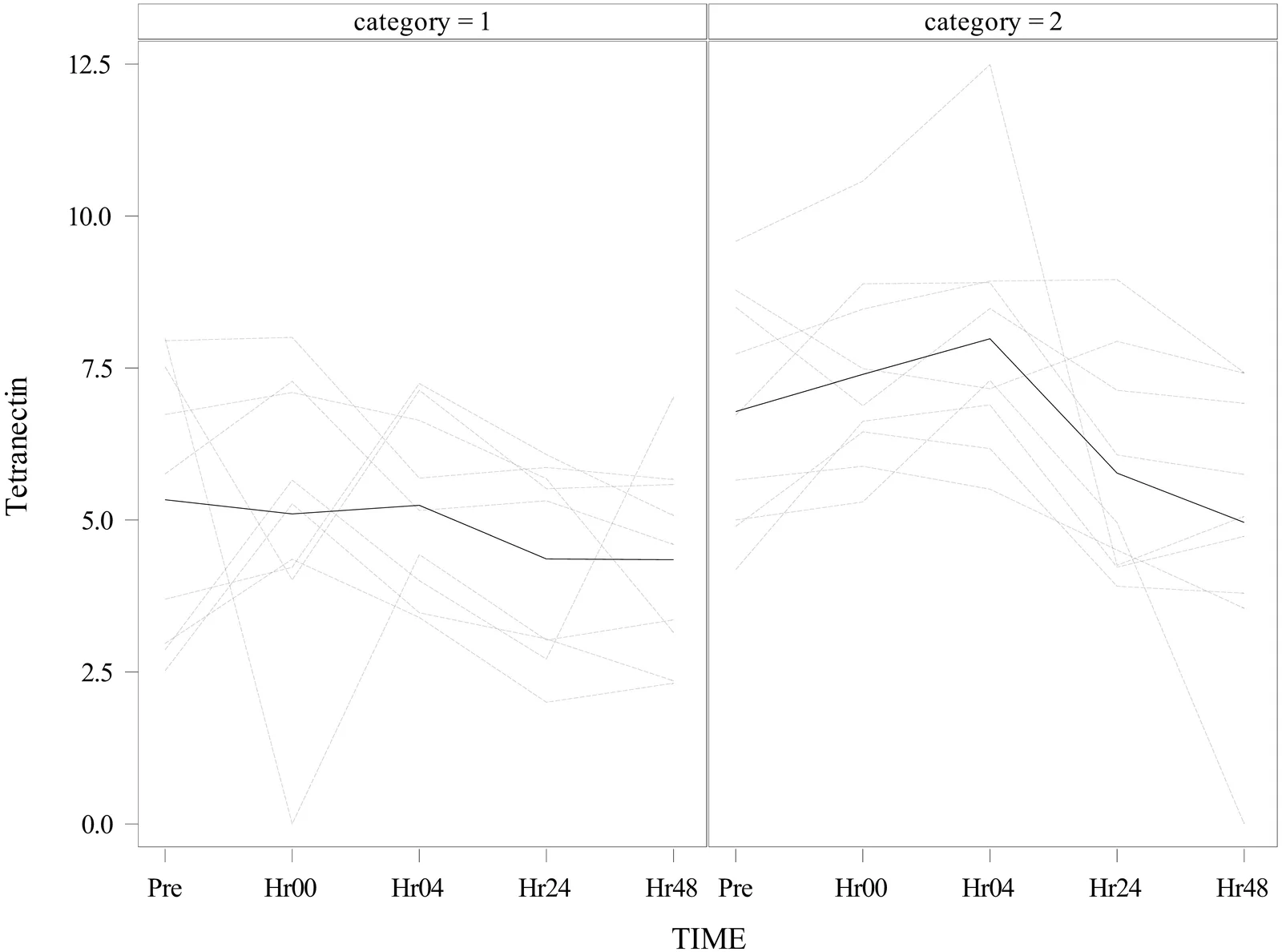
Graphical trend in Tetranectin levels in patients following cardiopulmonary bypass, stratified by duration of inotropic support. Category 1—lower tertile; Category 2—upper tertile. Pre, pre-op; hr, hours post-op.

To better understand the association of TN levels with the duration of inotropic support, we divided our cohort into tertiles based on their duration of inotropic support. Logistic regression analyses were performed, and we compared the upper (*n* = 9) and lower (*n* = 9) tertiles in relation to the trend in TN levels, which indicated a higher trend in TN levels in patients with a longer duration of inotropic support (Figure 2).

## 4. Discussion

LCOS is a known phenomenon following CPB and the timely recognition of this state remains critical. There have been various clinical and few biochemical biomarkers that have been studied as surrogates to predict LCOS. The two most utilized serum biomarkers are lactate and S_c_VO_2_ [14,20,21]. Serum lactate is the bioproduct of anaerobic metabolism and elevated serum lactate is used at bedside as a measure of inadequate oxygen delivery and end organ injury. S_c_VO_2_ on the other hand can be impacted by pulmonary venous desaturation, intracardiac shunting, anemia and low FiO_2_, all of which can decrease oxygen delivery independent of cardiac output [22]. Although these two biomarkers help in the diagnosis of the LCOS, a new serum biomarker that can predict the LCOS earlier in the clinical course can improve critical monitoring and outcomes.

Our study is the first to report TN as a potential prognostic biomarker for impaired cardiac output in neonates following complex cardiac surgery utilizing CPB such as Norwood operation. TN level at 4 h following surgery was significantly associated with longer duration of inotropic support during their acute post-operative course, while BNP levels did not show any correlation. This finding is important clinically as higher TN levels at 4 h following surgery can identify patients at risk for LCOS and potentially enable better perfusion targeted therapy. Duration of inotropic support is an imperfect surrogate for LCOS because it may be influenced by institutional practice, clinician preference and hemodynamic targets. Therefore, the observed association between TN and inotropic duration requires further investigation and cannot be interpreted as direct proof that TN identifies LCOS.

Prior studies of adult patients with heart failure reported variable levels of TN and its association with heart failure severity [16,23]. Panagiotopoulos et al. reported an increasing trend of TN on the first and third post-op day following CPB [17]. However, the same study reported that patients with heart failure have lower levels of TN compared to healthy individuals. CPB is known to trigger several inflammatory mechanisms including the coagulation cascade and the complement system that lead to endothelial damage and play a role in myocardial reperfusion injury following CPB [24].

TN is a plasminogen binding protein encoded by the CLEC3B gene and promotes the activation of plasminogen to regulate proteolytic processes. TN has been considered to play a role in tissue remodeling as well [25,26]. This is facilitated by its ability to bind ECM components such as fibrin and plasminogen, stimulate proteolytic activation of proteases and growth factors and regulate ECM proteolysis [27].

TN is thought to have an important protective role in myocardial ischemia. A biomarker analysis found that reduced circulating levels of TN are significantly associated with all-cause mortality and cardiovascular disease [28]. Another study noted that following an acute myocardial infarction, circulating TN levels were significantly reduced in the first three hours following the event [29]. TN overexpression has been shown to lead to increased cell viability and inhibit cellular apoptosis through a P13/Akt pathway [17,25]. While adult heart failure studies have reported associations between lower circulating TN and adverse cardiovascular outcomes, our pilot study data in this neonatal post-CPB cohort suggests that higher TN concentrations were associated with longer duration of inotropic support. The directionality and biological significance of TN in neonates after CPB remain unestablished. These findings may reflect differences between chronic adult heart failure biology and acute neonatal post-operative inflammatory, endothelial or tissue-remodeling responses. Mechanistic studies and pediatric reference ranges are needed before the physiological role of TN in this setting can be inferred.

## 5. Limitations

Our study is the first to explore TN as a potential biomarker for its utility in the prognostication of LCOS; however, there are several important limitations. This was a retrospective, single-center pilot study involving only 26 neonates, and the small sample size limits statistical power and generalizability of the findings. As only 18 patients were included in the tertile-based logistic regression analysis (the upper and lower tertile), we were unable to adjust for many covariates, and the AUC estimate from the tertile-based analysis may be optimistic because of the small sample size and availability of an independent validation cohort. Because of the pilot sample size, ROC analyses comparing the upper and lower tertiles of inotropic support were performed as exploratory analyses only. These analyses were not internally or externally validated and further investigation is necessary to verify if similar patterns are observed.

No a priori or post hoc power analyses were performed. This retrospective single-center pilot study was intended to describe serial TN concentrations and generate hypotheses regarding their association with post-operative clinical surrogates of LCOS following the Norwood procedure. The small sample size increases the risk of Type II error; therefore, the lack of association between TN and lactate clearance should not be interpreted as definitive evidence of no relationship and, similarly, observed associations with inotropic support should be considered exploratory and require prospective validation.

Serum samples were available only at predefined time points. Consequently, the temporal profile of TN between 4 and 24 h after CPB remains unknown, and the true peak concentration may have occurred between sampling intervals.

We did not perform TN stability testing across storage duration or repeated freeze–thaw cycles, nor could we measure TN in dialysate given the retrospective observational nature of this study. Therefore, effects of long-term storage, freeze–thaw exposure, and peritoneal dialysis on measured TN concentrations cannot be excluded.

Our cohort included only patients who are critically ill with no healthy cohort. Our investigation addresses changes in TN in sick neonates post-CPB and may not be extrapolated to other hypoperfusion states, other pediatric cardiac surgical populations, or non-cardiac causes of tissue hypoperfusion. At this point, we do not understand the biological role of this protein in healthy neonatal or pediatric subpopulations, which can limit our conclusion of the protective and prognostic role of TN. Future studies including healthy controls are needed to define age- and weight-specific reference ranges and to establish clinically meaningful threshold values for TN.

Additionally, although BNP was measured concurrently, the study was not powered to perform detailed comparative analyses between BNP and TN. Larger studies are required to determine whether TN offers incremental prognostic value beyond established biomarkers or whether a multi-marker strategy may improve risk stratification. Finally, TN assays are not currently available for routine clinical use. Therefore, the feasibility, cost-effectiveness, and practical implementation of TN testing in clinical practice remains uncertain at present.

## 6. Conclusions

In this pilot cohort of neonates undergoing the Norwood procedure, higher levels of serum TN, particularly at 4 h following CPB, were associated with a longer duration of post-operative inotropic support. These findings suggest that TN may serve as a promising biomarker of post-operative hemodynamic impairment and LCOS severity in this high-risk population. Larger prospective studies are needed to validate these findings, define clinically relevant TN thresholds, and determine whether TN provides incremental prognostic value beyond established biomarkers such as BNP.

## Figures and Tables

**Table 1:** Patient demographics and important clinical outcomes.

Patient Characteristics	*n* = 26
Age at time of surgery (days)	7.00 (5.75, 9.00)
Weight at time of surgery (kg)	3.21 (2.68, 3.40)
Gender (female), *n* (%)	14 (54)
HLHS (yes), *n* (%)	23 (88)
Norwood/Sano (yes), *n* (%) *	25 (96)
CPB (minutes)	142 (133, 164)
Aortic cross clamp (minutes)	58.8 (53.7, 71.0)
DHCA (minutes) **	23.5 (6.7, 52.5)
**Clinical Outcomes**	
Survival to discharge (yes), *n* (%)	24 (92)
Length of hospital stay (days)	37.5 (27.9, 53.9)
Length of mechanical ventilation (h)	129.8 (73.4, 210.7)
Duration of open sternum (h)	44.1 (41.1, 66.8)
ECMO (Yes), *n* (%)	4 (15)
Duration of inotropic support (h)	91.6 (71.9, 144.3)
Time to clear lactate (h)	25.0 (16.8, 41.7)
CRRT > 48 h (yes), *n* (%)	18 (69)
Duration of CRRT (h)	66.0 (40.5, 136.5)

Data presented as median (interquartile range). * Only one patient had Norwood and Blalock–Taussig shunt. ** Missing data: Only 16 patients information included. HLHS, hypoplastic left heart syndrome; CPB, cardiopulmonary bypass; DHCA, deep hypothermic cardiac arrest; CRRT, continuous renal replacement therapy.

**Table 2:** Descriptive analysis of both TN and BNP levels at several time points, *n* = 26.

BNP (ng/mL)	Mean ± SD	Median (IQR)
**Time point**		
Pre-op	11.195 ± 6.629	9.188 (6.30, 16.38)
Post-op 0 h	6.266 ± 5.760	4.135 (2.16, 11.55)
Post-op 4 h	14.727 ± 6.923	15.784 (9.19, 21.03)
Post-op 24 h	8.478 ± 5.052	7.409 (5.17, 13.96)
Post-op 48 h	4.184 ± 2.704	3.476 (2.57, 5.25)
**TN (mcg/mL)**		
**Time point**		
Pre-op	5.770 + 2.080	5.706 (4.36, 7.89)
Post-op 0 h	6.113 + 2.103	6.378 (5.27, 7.44)
Post-op 4 h	6.416 + 2.440	6.372 (5.24, 7.28)
Post-op 24 h	4.879 + 1.872	4.513 (3.98, 6.02)
Post-op 48 h	4.530 + 1.787	4.664 (3.40, 5.73)

TN, Tetranectin; BNP, B-type natriuretic peptide; SD, standard deviation; IQR, interquartile range.

**Table 3:** Descriptive analysis of several clinical indices for low cardiac output state.

Variables	Mean ± SD *n* = 26	Median (IQR) *n* = 26
**VIS**		
Pre-op	0 ± 0	0 (0, 0)
Post-op 0 h (*n* = 25)	12.64 ± 4.59	14.15 (9.00, 16.30)
Post-op 4 h	12.03 ± 3.90	12.3 (9.3, 14.0)
Post-op 24 h	10.18 ± 3.98	9.0 (7.7, 11.7)
Post-op 48 h	8.80 ± 4.11	7.6 (5.0, 12.0)
**NIRS**		
Pre-op	55.92 ± 11.78	57 (48, 62)
Post-op 0 h (*n* = 25)	61.16 ± 17.94	63.0 (46.5, 75.5)
Post-op 4 h	58.54 ± 12.27	57.5 (51.0, 69.0)
Post-op 24 h	60.69 ± 9.30	62 (57, 66)
Post-op 48 h	57.85 ± 12.94	55 (50, 66)
**S_c_VO_2_**		
Pre-op (*n* = 10)	66 ± 9.9	64 (59, 77)
Post-op 0 h (*n* = 24)	56 ± 10.6	58 (49, 64)
Post-op 4 h (*n* = 25)	55 ± 12.5	56 (46, 62)
Post-op 24 h (*n* = 22)	57 ± 7.5	57 (52, 63)
Post-op 48 h (*n*= 20)	55 ± 7.4	55 (49, 60)
**Lactate (mmol/L)**		
Pre-op	1.82 ± 1.48	1.45 (1.05, 1.95)
Post-op 0 h	8.23 ± 3.22	7.8 (6.6, 9.8)
Post-op 4 h	5.93 ± 3.78	5.2 (3.3, 6.9)
Post-op 24 h	1.98 ± 0.60	1.95 (1.5, 2.4)
Post-op 48 h	1.52 ± 0.53	1.4 (1.1, 1.7)
**Percent fluid overload**		
Post-op 24 h	4.88 ± 6.68	3.82 (0.39, 8.27)
Post-op 48 h	−1.35 ± 3.58	−0.34 (−4.70, 1.44)

SD, standard deviation; VIS, vasoactive inotrope score; NIRS, near-infrared spectroscopy; S_c_VO_2_, central venous oxygen saturation.

**Table 4:** Results from univariate and multivariable survival weibull model for time to lactate clearance and duration of inotropic support.

Predictor	Relative Duration with CI	*p*-Value
**TN (mcg/mL)**	**Relative Duration of Time to Lactate Clearance**	
0 h	0.97 (0.84, 1.11)	0.617
4 h	0.97 (0.83, 1.14)	0.753
	**Relative Duration of Inotropic Support**	
0 h	1.14 (1.03, 1.25)	0.0086
4 h	1.12 (1.01, 1.23)	0.0285
**TN (mcg/mL) adjusted for BNP (ng/mL)**	**Relative Duration of Time to Lactate Clearance when Adjusted for BNP**	
0 h	0.99 (0.93, 1.07)	0.945
4 h	1.01 (0.97, 1.06)	0.548
	**Relative Duration of Inotropic Support when Adjusted For BNP**	
0 h	1.13 (1.03,1.25)	0.0095
4 h	1.12 (1.01, 1.24)	0.0245

Relative estimated time to lactate clearance and estimated duration of inotropic support is for 1 unit increase in TN. TN, Tetranectin; CI, confidence interval; BNP, B-type natriuretic peptide.

## Data Availability

The data presented in this study are available on request from the corresponding author.
